# Pedestrian Re-Identification Based on Fine-Grained Feature Learning and Fusion

**DOI:** 10.3390/s24237536

**Published:** 2024-11-26

**Authors:** Anming Chen, Weiqiang Liu

**Affiliations:** Tsinghua Shenzhen International Graduate School, Tsinghua University, Shenzhen 518055, China; liuwq@tsinghua-sz.org

**Keywords:** pedestrian re-identification, token learning, multimodal fusion

## Abstract

Video-based pedestrian re-identification (Re-ID) is used to re-identify the same person across different camera views. One of the key problems is to learn an effective representation for the pedestrian from video. However, it is difficult to learn an effective representation from one single modality of a feature due to complicated issues with video, such as background, occlusion, and blurred scenes. Therefore, there are some studies on fusing multimodal features for video-based pedestrian Re-ID. However, most of these works fuse features at the global level, which is not effective in reflecting fine-grained and complementary information. Therefore, the improvement in performance is limited. To obtain a more effective representation, we propose to learn fine-grained features from different modalities of the video, and then they are aligned and fused at the fine-grained level to capture rich semantic information. As a result, a multimodal token-learning and alignment model (MTLA) is proposed to re-identify pedestrians across camera videos. An MTLA consists of three modules, i.e., a multimodal feature encoder, token-based cross-modal alignment, and correlation-aware fusion. Firstly, the multimodal feature encoder is used to extract the multimodal features from the visual appearance and gait information views, and then fine-grained tokens are learned and denoised from these features. Then, the token-based cross-modal alignment module is used to align the multimodal features at the token level to capture fine-grained semantic information. Finally, the correlation-aware fusion module is used to fuse the multimodal token features by learning the inter- and intra-modal correlation, in which the features refine each other and a unified representation is obtained for pedestrian Re-ID. To evaluate the performance of fine-grained features alignment and fusion, we conduct extensive experiments on three benchmark datasets. Compared with the state-of-art approaches, all the evaluation metrices of mAP and Rank-K are improved by more than 0.4 percentage points.

## 1. Introduction

With the development of electronic devices, the importance of video surveillance to social security is becoming increasingly prominent. Pedestrian re-identification (Re-ID) is used to re-identify pedestrians across non-overlapping cameras in surveillance systems, which is important to video surveillance. According to the application scenarios, pedestrian Re-ID can be categorized into image-based methods [1,2,3,4] and video-based methods [5,6,7]. Image-based pedestrian Re-ID aims to retrieve given pedestrians across different images, while video-based pedestrian Re-ID aims to retrieve given pedestrians across different videos. Different from image-based pedestrian Re-ID, video-based pedestrian Re-ID should utilize a sequence of image frames as inputs rather than a single image, which introduces additional motion cues besides the appearance observation. Therefore, video-based pedestrian Re-ID faces more challenges. Although that information is conducive to pedestrian Re-ID, it also introduces more noise and misalignments, such as that the appearance and motion might be reflected by heterogeneous features and there are more complicated backgrounds and occlusion scenarios. By analyzing the motion patterns of individuals, gait recognition offers unique and complementary information to appearance-based features, particularly in scenarios with limited visibility of the face or body. Therefore, how to effectively fuse the different views of features is an interesting problem in video-based pedestrian Re-ID.

There are already some research studies on video-based pedestrian Re-ID. These works exploit different clues from video for pedestrian Re-ID, such as spatial, temporal and spatial–temporal information. For example, some works adapt a spatial feature extractor to obtain attentive features from spatial dimensions, such as extracting aligned local features from multiple images with a set of diverse spatial attention networks [4]. Some other works exploit temporal relations in the temporal sequence with temporal learning networks, such as recurrent neural network (RNNs) [8] and Long Short-Term Memory (LSTM) [9]. Meanwhile, some works try to exploit both the spatial and temporal information, such as a multi-scale 3D network to learn multi-scale spatial-temporal cues [10] and trigeminal transformers on the spatial, temporal, and spatial–temporal dimensions [11]. Though these models achieve great success, they mainly learn a global representation from each view, which is not effective in reflecting the various and fine-grained characteristics of the pedestrian, such that each pedestrian has static appearance characteristics and also moves in the camera scene. Therefore, the model should learn different modalities of fine-grained features from different views of the video scene to obtain a more comprehensive and effective representation.

However, it is still challenging to fuse the fine-grained features learned from different views of the video scene. First, to more effectively reflect the characteristics of different views, we should learn different modalities of features corresponding to different views from the raw video instead of different mappings of the same features, such as [11]. Secondly, the different modalities of features are heterogeneous; an effective feature fusion method should be able to capturing fine-grained and cross-modal relations. Thirdly, the learned features should effectively capture fine-grained semantic and discriminative information. How to leverage this information simultaneously in representation learning is still a problem.

There are many methods proposed to exploit the cross-modal relationships between different modalities of features. These works can be categorized into representation-based and interaction-based approaches, which are mainly to learn and fuse different modalities of representation to discover the cross-modal correlation. Most of these methods follow two typical paradigms: the global alignment paradigm and the token fusion paradigm. In the global alignment paradigm, representation-based methods initially represent each modality through a sub-network containing modality-specific encoders to embed different modalities into global representations. Subsequently, they incorporate additional constraints in the learning objective to regularize the different modality representations, such as multi-task learning [12,13], mutual information maximization [14], tensor canonical correlation analysis [15,16], self-supervised learning [17,18,19], and so on. As for the token fusion paradigm, an interaction-based method is used to design sophisticated fusion architectures to facilitate token interactions between different modalities, such as attention-based [20,21], graph-based [22], and translation-based fusion [23]. However, there are still several problems in these works.

Firstly, the representation-based methods mainly encode each modality into a global representation, which is not effective in capturing fine-grained information. Meanwhile, interaction-based methods may overlook the redundant information contained in the original modality sequences. However, not all the features from different modalities are related to the target task, and potentially irrelevant information in heterogeneous modalities interferes with the target task. Secondly, the current methods mainly align different modalities of features at the global level, which is not effective in learning the fine-grained semantic correlation between different modalities. For example, global alignment methods apply weak contrasts to different modality representations, which is insufficient to maximize dependencies between the tokens of different modalities. Meanwhile, some token fusion methods model the interaction between different tokens directly and indiscriminately, which neglects the alignment between different modalities of tokens and thus is not effective in learning discriminative information.

To address these problems, we propose a novel multimodal token-learning and alignment (MTLA) framework for video-based pedestrian re-identification. Our primary objective is to effectively combine the complementary information from different views of the video, which significantly enhances the discriminative power of the learned features. Specifically, we learn multimodal and fine-grained features from the visual appearance and gait views, and then align them at the token level before feature fusion. The two modalities of fine-grained features which are denoted as tokens are learned from raw video data, which is more effective in capturing comprehensive information of the pedestrian in video. Similar to the word tokens in text data, the token feature is more abstract than the raw feature. Compared to the traditional global representation, the token feature is more effective in capturing fine-grained semantic information and also more effective in reducing the semantic gap between different modalities. Subsequently, a token-level cross-modal alignment is proposed to align different modalities of features via fine-grained contrastive learning. This method has two attractive properties. One is filtering out unnecessary information by maximizing the mutual information between two modalities, and the other one is performing a fine-grained alignment to facilitate multimodal interaction. Finally, based on the aligned fine-grained representations, a correlation-aware multimodal fusion method is proposed to learn the latent correlation between different modalities. It learns the cross-modal correlation based on hyper-attention and cross-attention mechanisms to generate a consistent and complementary multimodal representation.

In summary, we address the major limitations of the existing video-based person re-identification approaches from two aspects. First, the existing approaches fuse multimodal features at the low level. There exists a semantic gap between them and different types of features in a different semantic granularity. We learn a more abstract feature, i.e., tokens, to reduce the semantic gap and semantic representation differences between different modalities. Second, the existing approaches align cross-modal features at a global level, which is not effective for learning the fine-grained semantics from each. We propose to align the different modalities of features at the token level. The contributions of this paper can be summarized as follows:

1. We propose a novel multimodal fine-grained feature-learning and fusion model for video-based pedestrian re-identification, which exploits both the appearance and motion features to learn a more comprehensive representation. To the best of our knowledge, this is the first attempt to combine fine-grained appearance and motion features for pedestrian Re-ID;
2. We design a token-level alignment to learn discriminative and important information from different modalities of the features, and then the inter-modal relation is learned by a cross-attention method to fuse the different modalities of features;
3. We conduct extensive experiments on three benchmark datasets to evaluate the performance of the proposed method, and all the evaluation metrices of mAP and Rank-K are improved by more than 0.4 percentage points.

## 2. Related Works

With the development and popularity of digital technology, pedestrian re-identification has been extensively studied in recent years. Research on pedestrian re-identification can be roughly categorized into two groups: pedestrian Re-ID based on static images [3] and pedestrian Re-ID based on video sequences [5,24,25,26,27]. Due to the urgent need for video matching in intelligent control applications, video-based pedestrian Re-ID has gained more and more attention. Compared with static images, videos contain more views which are worthy of observing, such as the personal appearance view and motion view, and also the video data is spatially and temporally organized. Therefore, video-based Re-ID is more challenging than image-based Re-ID. Therefore, existing works focus on capturing spatial information, temporal information, and multiple views.

### 2.1. Uni-Modal Feature-Based Approaches

The first groups of works mainly attempt to extract attentive features from the spatial dimension [4,5,6,28,29]. For example, Li et al. [5] propose to extract the aligned spatial features from a sequence of images using a spatial attention mechanism. Zhao et al. [28] learns the frame-wise features for various attribute-aware representations in the spatial dimension. On the other hand, some works concentrate on learning from the temporal dimension [9,24,30,31]. For example, Mclaughlin et al. [8] adapt recurrent neural networks (RNNs) across frames to learn from the temporal dimension. A refining recurrent unit is proposed by Liu et al. [9] to integrate frames in the temporal sequence. Li et al. [32] propose to extract a global–local temporal representation for pedestrian Re-ID. A Context-Sensing Attention Network (CSA-Net) is proposed to improves both the frame feature extraction and temporal aggregation steps by Wang et al. [33]. Another work integrates a person attributes feature and scene attributes feature with an attention mechanism to address the problem of occlusion in video Re-ID [12].

### 2.2. Multimodal Feature-Based Approaches

With the development of transformer and its application [34,35,36,37,38], there are many works on learning spatial–temporal features, which is also combined with a CNN model. For example, to preserve the appearance features, Gu et al. [25] propose a specific 3D convolutional network to exploit temporal information and address the appearance destruction problem. Li et al. [10] propose a two-stream convolutional network to learn from both the spatial and temporal dimensions. With the development of vision transformer learning [34,39], a trigeminal network is proposed by Liu et al. [11] to transform raw video data into a spatial, temporal, and spatial–temporal feature space in three different views. The authors of [39] couple CNNs and transformers to extract two kinds of visual features, and a hierarchical temporal aggregation is proposed to progressively capture inter-frame dependencies and encode temporal information. Tang et al. [40] propose a Multi-Stage Spatial–Temporal Aggregation Transformer to extract local attributes and global identity information by leveraging spatial and temporal clues. Hou et al. [27] aim to decrease the influence of occlusion by proposing a spatio-temporal completion network. Zang et al. [21] propose a multi-direction and multi-scale pyramid in transformer for video-based pedestrian retrieval.

Different from these works, in this paper, we design a dual network to learn different modalities of fine-grained features from different views of the video. Then, the different modalities of tokens are aligned based on fine-grained interaction. Therefore, our model can capture more discriminative information about pedestrians. Finally, the different modalities of tokens are fused based on the correlation to learn the presentation of the pedestrian. The advantage of the proposed method is that it learns the fine-grained token features instead of a global representation from the visual input, which are similar to the word tokens in a text document. Compared with the raw features or global representation, the token features are more effective in reflecting semantics and discriminative information, and they are also reinforced by cross-modal alignment via contrastive learning.

## 3. Proposed Model

In this section, the proposed model is detailed. We first present an overview of the proposed model, and the key modules are elaborated in the following subsections.

### 3.1. Overview

Given an annotated video dataset $S=\{(V_{1},y_{1}),(V_{2},y_{2}),\ldots ,(V_{N},y_{N})\}$, where *V_i_* denotes a tracklet and consists of a series of images, i.e., $V_{i}=\{I_{i}^{\left(1\right)},I_{i}^{\left(2\right)},\ldots ,I_{i}^{\left(n\right)}\}$, and *y_i_* is the ground truth label of the corresponding identity, the goal of video-based person re-identification is to learn a feature embedding for each tracklet, where the representations of the same identity are closer than that of different identities. To learn the representation, there are two types of object functions, i.e., classification loss and distance metric learning loss. The classification loss is used to classify the same pedestrian into the same category. As for the distance metric learning loss, various versions of the triplet loss [41] are used to learn the representation for each tracklet.

There are also some works on combining multiple features for pedestrian re-identification. One category of these methods learns the visual features from both the temporal and spatial dimensions, and then the two types of features are fused with the attention mechanism for re-identification [10,11]. The other category of methods extracts different features with different models, such as transformer and CNNs [39]. Then, the two types of features are fused by exploiting the hierarchical correlation between them. However, these methods extract different features by different models, where redundant information might be generated between these features. The different features are fused based on a bipartite relation, which is not effective in reflecting the various relations between pedestrian features, such as appearance, background, movement, and illumination.

**Figure 1:**
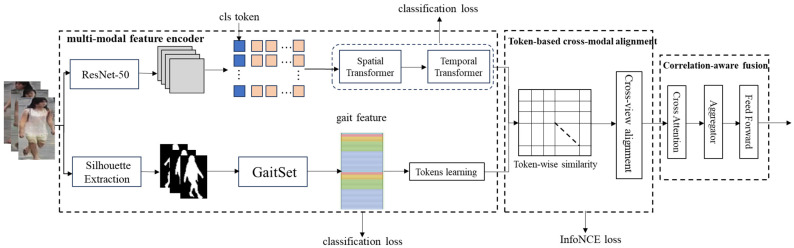
Framework of MTLA, which consists of three modules; i.e., a multimodal feature encoder is used to learn fine-grained and discriminative features from the visual appearance and gait views, token-based cross-modal alignment is used to align the tokens based on the contrastive learning method, and correlation-aware fusion is used to fully integrate the two types of features to obtain an effective representation via cross-attention and aggregator.

To effectively learn discriminative features from different modalities for video-based pedestrian re-identification, we propose a multimodal learning model with a dual network (MTLA) to fuse the gait and visual appearance feature. As shown in Figure 1, the framework of the model mainly consists of three modules, i.e., a multimodal feature encoder, token-based cross-modal alignment, and correlation-aware fusion. The multimodal feature encoder is used to learn fine-grained and discriminative features from the vision appearance and gait modalities of the tracklet. The token-based cross-modal alignment is used to align the tokens by exploiting their cross-modal relation with a contrastive learning method [42,43,44]. The correlation-aware fusion module is then used to fully integrate the aligned tokens to obtain an effective representation via cross-attention and aggregator.

### 3.2. Multimodal Feature Encoder

We first extract the gait feature and visual features using two extractors. For the visual features, we should also capture the spatial and temporal information of the frames in each tracklet. Specifically, we use the transformer [34] method to encode the visual features. Beside the visual features, the gait feature is also important to reflect the action and movement characteristics. These two types of features are complementary to each other.

#### 3.2.1. Visual Appearance Token Learning

Similar to [45], the input tracklet is formulated as $X_{v}\in {\mathbb{R}}^{h\times w\times 3\times n}$, consisting of *n* RGB frames of size h×w. Each frame is decomposed into m non-overlapping patches with a size s×s, and the total number of patches in a frame is $m=hw/s^{2}$. Then, these patches are flattened into vectors $x_{\left(l,t\right)}\in {\mathbb{R}}^{3s^{2}}$, where l=1,…, m denotes the spatial location and t=1,…, n denotes the index of the frame.

First, each patch is mapped into a vector $z_{\left(l,t\right)}^{0}\in {\mathbb{R}}^{d}$ as follows:

(1) $$z_{\left(l,t\right)}^{0}=W^{0}x_{\left(l,t\right)}^{0}+e_{\left(l,t\right)}^{pos}$$

where $W^{0}\in {\mathbb{R}}^{d\times 3s^{2}}$ is a matrix to be learned and $e_{\left(l,t\right)}^{pos}$ is a positional embedding to encode the position of each patch. The resulting vectors $z_{\left(l,t\right)}^{0}=W^{0}x_{\left(l,t\right)}^{0}+e_{\left(l,t\right)}^{pos}$ (l=1,…, m and t=1,…, n) are then input to the transformer, and another vector $z_{\left(0,0\right)}^{0}\in R^{d}$ is added in the first position to represent the embedding of the classification token.

**Figure 2:**
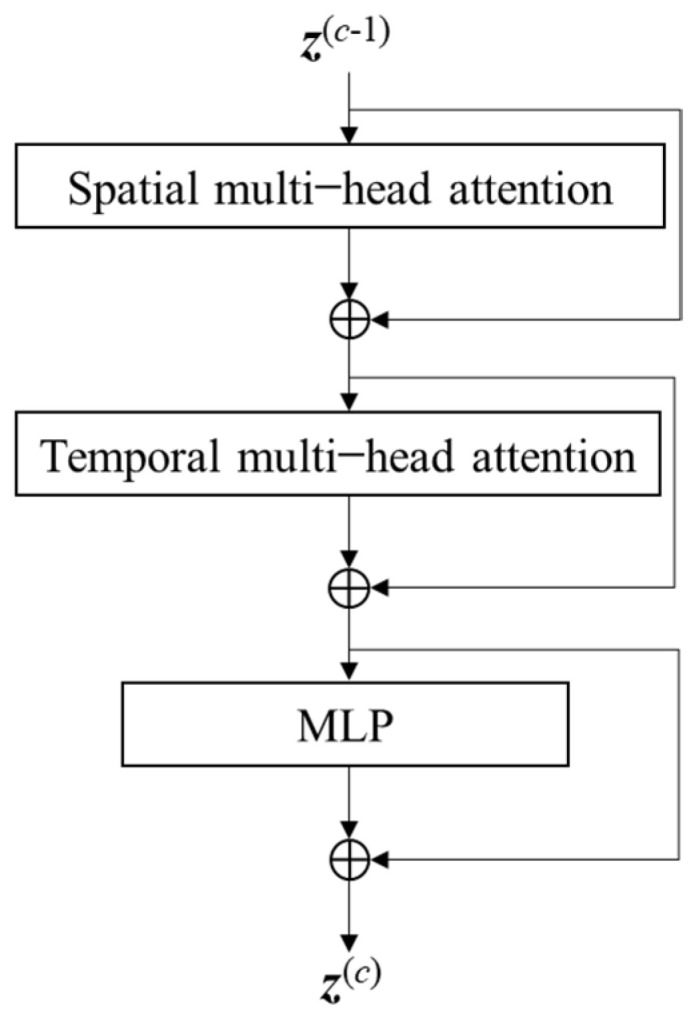
The attention block.

The transformer consists of *C* encoding blocks, and the structure of each block is shown in Figure 2. Since the tracklet is composed of sequential frames, the tokens are correlated in both the spatial dimension in each frame and the temporal dimension in the sequence. Therefore, the attention is calculated over the temporal and spatial dimension, respectively, to learn the temporal and spatial correlation between the features. Specifically, at each block, we first compute the spatial multi-head attention, and then the temporal multi-head attention is computed. Finally, the output is sent into an MLP.

1. Spatial Attention

In the *c*-th block, the multi-head attention is first calculated in the spatial dimension. To perform the spatial attention calculation, a triplet term of <query, key, value> is calculated for each patch from the output of the preceding block as follows:

(2) $$q_{\left(l,t\right)}^{\left(c,a\right)}=W_{Q^{\mathrm{space}}}^{\left(c,a\right)}LN(z_{\left(l,t\right)}^{\left(c-1\right)})\in {\mathbb{R}}^{D_{h}}$$

(3) $$k_{\left(l,t\right)}^{\left(c,a\right)}=W_{K^{\mathrm{space}}}^{\left(c,a\right)}LN(z_{\left(l,t\right)}^{\left(c-1\right)})\in {\mathbb{R}}^{D_{h}}$$

(4) $$v_{\left(l,t\right)}^{\left(c,a\right)}=W_{V^{\mathrm{space}}}^{\left(c,a\right)}LN(z_{\left(l,t\right)}^{\left(c-1\right)})\in {\mathbb{R}}^{D_{h}}$$

where LN(.) is the LayerNorm [46] and *a* is an index of the heads in the multi-head attention mechanism. By setting the total number of heads as *A*, we can obtain the dimension of each head $D_{h}=d/A$.

The spatial attention weights are calculated with dot product. For the query patch (*l*, *t*), the attention weight is calculated as follows:

(5) $$\alpha_{\left(l,t\right)}^{(c,a)\mathrm{space}}=\mathrm{Soft}\max\left(\frac{q_{\left(l,t\right)}^{{\left(c,a\right)}^{T}}}{\sqrt{D_{h}}}\cdot \left[k_{\left(0,0\right)}^{\left(c,a\right)}{\left\{k_{\left(l,t^{\prime }\right)}^{\left(c,a\right)}\right\}}_{t^{\prime }=1,\ldots ,n}\right]\right)$$

With the spatial attention, the temporal encoding at the *c*-th block can be obtained by the weighted summarization and concatenation operations. First, we sum the value vectors using the attention weights from each head as follows:

(6) $$z_{\left(l,t\right)}^{0}=W^{0}x_{\left(l,t\right)}^{0}+e_{\left(l,t\right)}^{pos}$$

Then, the vectors learned from all the heads are concatenated, to be projected with a residual connection as follows:

(7) $${z^{\prime }}_{\left(l,t\right)}^{(c)\mathrm{space}}=W^{c}\left[\begin{array}{c} s_{\left(l,t\right)}^{\left(c,1\right)}\\ \vdots \\ s_{\left(l,t\right)}^{\left(c,A\right)} \end{array}\right]+z_{\left(l,t\right)}^{\left(c-1\right)}$$

2. Temporal Attention

As shown in Figure 2, the output ${z^{\prime }}_{\left(l,t\right)}^{(c)\mathrm{space}}$ of the spatial attention operation is then sent into the temporal attention calculation. That is, new key/query/value vectors are obtained from ${z^{\prime }}_{\left(l,t\right)}^{(c)\mathrm{space}}$. Specifically, we first learn the query/key/value matrices $\left\{W_{Q^{time}}^{\left(c,a\right)},W_{K^{time}}^{\left(c,a\right)},W_{V^{time}}^{\left(c,a\right)}\right\}$ and obtain the query/key/value vectors like Equations (2)–(4). Then, the temporal attention is calculated, like Equation (5). With the attention weights, we can obtain the weighted sum of the values and the concatenation value ${z^{\prime }}_{\left(l,t\right)}^{(c)time}$ like Equations (6) and (7). Finally, the resulting vector ${z^{\prime }}_{\left(l,t\right)}^{(c)time}$ is sent into the MLP as follows:

(8) $$z_{\left(l,t\right)}^{\left(c\right)}=MLP\left(LN({z^{\prime }}_{\left(l,t\right)}^{(c)time})\right)+{z^{\prime }}_{\left(l,t\right)}^{(c)time}$$

3. Classification Loss

In the spatial attention and temporal attention steps, the feature-learning process is unsupervised. Therefore, the learned features might not be effective in reflecting personal appearance characteristics. To learn more discriminative features, we add a classifier to the final appearance embedding obtained from the class token representation $z_{\left(0,0\right)}^{\left(C\right)}$ as follows:

(9) $$z^{p}=LN\left({z^{\prime }}_{\left(0,0\right)}^{\left(C\right)}\right)$$

The cross-entropy loss is used to learn the classifier, and then the learned tokens are forced to focus on the human information to discriminate the input. The tokens learned from the transformer module for the *i*-th tracklet are serialized as $Z_{i}^{v}=\{z_{\left(l,t\right)}^{\left(C\right)}|l=1,\ldots ,m,t=1,\ldots ,n\}$.

#### 3.2.2. Gait Token Learning

The gait features mostly reflect personal movement characteristics, which can supplement the appearance features. Since the gait feature is unrelated with the background, an individuality-preserving silhouette extraction [47] is first performed on the tracklet. Then, the gait feature representation ***E_i_*** is learned from the gait silhouettes using the model GaitSet [48] as follows:

(10) $$E_{i}=H(G(F(\{x_{i}\}))$$

where $\left\{x_{i}\right\}$ denotes the sequence of silhouettes with four dimensions, i.e., set dimension, image channel dimension, image height dimension, and image width dimension, F(.) is a convolutional network to learn the frame-level features from each silhouette, G(.) is a permutation invariant function used to map a set of frame-level feature to a set-level feature, and function H is used to learn the discriminative representation from the set-level feature based on a structure called Horizontal Pyramid Mapping (HMP). Therefore, we take the feature map generated by MGP, and then it is also mapped into $\sum_{s=1}^{S}2^{s-1}$ features by HPM as the raw input of the gait modality.

However, the raw feature is high-dimensional and redundant. Therefore, we learn a set of refined tokens from the raw feature to obtain a more effective representation of the gait modality. That is, given the raw gait feature representation $E_{i}\in {\mathbb{R}}^{l\times d}$, we learn a set of tokens $G_{i}\in {\mathbb{R}}^{k\times d}$, where k≪l. To perform an aligning process, we set that the numbers of tokens learned from visual appearance and gait information are equal, i.e., k=n×m. First, the token-level relevance is calculated to learn the correlation between them, which is used to extract the fine-grained information from the gait modality. This process reduces the influence of irrelevant features that are less correlated with the tokens, and it is formulated as follows:

(11) $$G_{i}^{\prime }=Norm(G_{i}+CrossAtt(G_{i},E_{i},E_{i}))$$

(12) $$CrossAtt(G_{i},E_{i},E_{i})=\mathrm{soft}\max(\frac{G_{i}E_{i}^{T}}{\sqrt{d}})E_{i}$$

where $G_{i}$ is the token representation to be learned. Then, self-attention is used to learn the salient information of the gait tokens as follows:

(13) $$G_{i}^{\prime \prime }=Norm(G_{i}^{\prime }+SelfAtt(G_{i}^{\prime },G_{i}^{\prime },G_{i}^{\prime }))$$

(14) $$SelfAtt(G_{i}^{\prime },G_{i}^{\prime },G_{i}^{\prime })=\mathrm{soft}\max(\frac{G_{i}^{\prime }{G_{i}^{\prime }}^{T}}{\sqrt{d}})G_{i}^{\prime }$$

The final process is a position-wise feed-forward layer to generate the fine-grained representation $Z_{i}\in {\mathbb{R}}^{k\times d}$ as follows:

(15) $$Z_{i}^{g}=Norm(G_{i}^{\prime \prime }+FFN(G_{i}^{\prime \prime }))$$

(16) $$FFN(G_{i}^{\prime \prime })=ReLU(G_{i}^{\prime \prime }W_{g}^{\prime }+b_{g}^{\prime })W_{g}^{\prime \prime }+b_{g}^{\prime \prime }$$

This module facilitates the learned tokens to iteratively interact with the original features through the stacking of multiple learning layers. Therefore, redundant and noisy information can be gradually reduced, which preserves the fine-grained gait information. To make the learned tokens more discriminative and add the supervision information, we conduct mean pooling on the tokens to obtain a vector for pedestrian classification.

### 3.3. Token-Based Cross-Modal Alignment

The features of different modalities are learned separately, and they are heterogeneous and inconsistent. Therefore, it is necessary to learn the correlation and the complementary information between them to further improve the representation of different modalities. The existing works [49] on features fusion or alignment mainly process the tokens as a whole, which is not effective in capturing the fine-grained correlation between tokens. We perform the cross-modal alignment based on the token embeddings. First, we calculate the similarity between two modalities based on the tokens. Then, the two modalities are aligned with the contrastive learning method [42,43,44].

To obtain the similarity between two modalities, the token-wise similarity matrix between the two types of tokens is as follows:

(17) $$M=Z_{i}^{g}Z_{i}^{v}$$

The token-based similarity matrix $M\in {\mathbb{R}}^{k\times k}$ represents the matching scores between visual appearance and gait text tokens, Then, two rounds of attention pooling are conducted on the matrix to learn the consistent information between them. The initial pooling operation is designed to obtain the token-level similarity vector as follows:

(18) $$M_{v}=\sum_{i=1}^{k}\frac{\exp(M_{\left(*,i\right)}/\sigma )}{\sum_{j}^{k}\exp(M_{\left(*,j\right)}/\sigma )}M_{\left(*,i\right)}$$

where * denotes the sum of all elements in the corresponding axis and σ is the temperature parameter. $M_{v}\in {\mathbb{R}}^{k}$ denotes the dynamic similarity weights of gait tokens to the visual appearance modality. To derive the final fine-grained similarity score, a second pooling is then conducted on the token-level similarity scores $M_{v}$, represented as

(19) $$M_{gv}=\sum_{i=1}^{k}\frac{\exp(M_{v(*,i)}/\sigma )}{\sum_{j}^{k}\exp(M_{v(*,j)}/\sigma )}M_{v(*,i)}$$

where $M_{gv}\in {\mathbb{R}}^{1}$ is the final similarity score of the two modalities calculated based on the tokens, which also can be used to calculate the similarity of the two modalities of tokens from two tracklets. We define the aforementioned calculation process $M_{gv}\in {\mathbb{R}}^{1}$ as a similarity function $s(Z^{g},Z^{v})$. Then, the contrastive learning method is applied to align the two types of tokens by exploring the correlation between them. The main idea is that the final appearance tokens and the gait tokens of the same pedestrian should be more similar than that of different pedestrians. We maximize the agreement of tokens across different modalities in the common space. Therefore, the cross-modal tokens discrimination $L_{p\text{-}g}$ of appearance to gait and cross-modal tokens discrimination $L_{g\text{-}p}$ are formulated by the InfoNCE loss as follows:

(20) $$L_{v-g}=-\sum_{i=1}^{K}\log\frac{exp(s(Z_{i}^{v},Z_{i}^{g})/\upsilon )}{\sum_{j=1}^{N}1_{i\neq j}exp(s(Z_{i}^{v},Z_{j}^{g})/\upsilon )}$$

(21) $$L_{g-p}=-\sum_{i=1}^{K}\log\frac{exp(s(Z_{i}^{g},Z_{i}^{v})/\upsilon )}{\sum_{j=1}^{K}1_{i\neq j}exp(s(Z_{i}^{g},Z_{j}^{v})/\upsilon )}$$

where *K* is the mini-batch size, υ is the temperature coefficient and $Z_{i}^{v}$ and $Z_{i}^{g}$ are the feature vectors learned from different modalities of the same data. In contrast to global alignment methods, token-based cross-modal alignment captures a fine-grained alignment across different modalities of tokens. Therefore, it can improve the learning of complementary information from multimodal features by filtering out noise and redundant information.

### 3.4. Correlation-Aware Fusion

After multimodal feature learning, we obtain two types of features learned from each other. Then, we can fuse the two types of features for pedestrian re-identification. A simple way is to directly concatenate them. This method is not effective in capturing the pairwise interactions between the tokens. We apply a correlation-aware multimodal fusion module to learn the latent correlations across modalities, to obtain consistent and complementary multimodal representations. This module comprises three layers, i.e., the cross-attention layer, the aggregator layer, and the feed-forward layer.

The cross-attention operation that uses appearance tokens as the value is formulated as follows:

(22) $$CrossAtt(Z_{i}^{v},Z_{i}^{g})=softmax\left(\frac{Q_{i}^{v}K_{i}^{gT}}{\sqrt{d}}\right)V_{i}^{g}\;\begin{array}{l} Q_{i}^{v}=Z_{i}^{v}W^{Q}\\ K_{i}^{g}=Z_{i}^{g}W^{K}\\ V_{i}^{g}=Z_{i}^{g}W^{V} \end{array}$$

Then, the cross-attention $CrossAtt(Z^{g},Z^{v})$ using gait tokens as the value is also calculated as in the aforementioned formulation. Subsequently, the aggregation function is used to integrate them, and thus a multimodal representation that contains consistent information is obtained. We use element-wise addition to integrate the representations. The whole operation of the process can be calculated as follows:

(23) $$R_{i}=Norm(CrossAtt(Z_{i}^{v},Z_{i}^{g})+CrossAtt(Z_{i}^{g},Z_{i}^{v}))$$

Finally, a feed-forward layer is applied to facilitate optimization and obtain the fusion results as

(24) $${\hat{R}}_{i}=Norm\left(R_{i}+ReLU(R_{i}W_{1}+b_{1})W_{2}+b_{2}\right)$$

We stack multiple correlation-aware fusion layers to produce consistent and complementary multimodal representations. These representations consider the latent correlations across modalities and mitigate the adverse effects of inconsistent information.

### 3.5. Pedestrian Re-ID

After the fusion process, the multimodal fusion representation consisting of a set of tokens is subject to mean pooling to obtain the final representation for pedestrian Re-ID. Particularly, we use the triplet loss to learn the distance metric. Then, the total training loss consists of several losses as follows:

(25) $$L_{total}=L_{st}+L_{gait}+L_{align}+L_{trip}$$

where $L_{st}$ is the classification loss of the spatial and temporal transformer on the visual appearance tokens, $L_{gait}$ is the classification loss used to learn the gait tokens, $L_{align}=L_{p-g}+L_{g-p}$ is the loss of the cross-modal alignment, and $L_{trip}$ is the triplet loss for distance metric learning on the fused representation. In the test process, the mean pooling of the fused representation is used to identify the pedestrian.

## 4. Experiments

We evaluate our model by comparing it with several related works in three datasets.

### 4.1. Datasets and Evaluation Metrics

To evaluate the performance of the proposed model, three widely used benchmarks are used to conduct the experiments, i.e., iLIDS-VID [50], PRID-2011 [51] and MARS [52], which are detailed in Table 1. iLIDS-VID and PRID-2011 contain videos taken by two cameras, and they are two small datasets. Specifically, there are 600 video tracklets of 300 identities in the dataset iLIDS-VID, while PRID-2011 contains about 400 tracklets of 200 identities from two non-overlapping cameras. MARS is a larger dataset which contains 20,478 tracklets of about 1261 identities. All the tracklets are captured by at least 2 cameras. To analyze the performance, the mean Average Precision (mAP) and Cumulative Matching Characteristic (CMC) table are used for evaluation in the dataset of MARS as previous works [4]. Since the datasets iLIDS-VID and PRID2011 have only one single correct match, only the metric of cumulative accuracy is adopted for evaluation.

### 4.2. Experiment Configuration

Each image in a tracklet is resized to 224 × 224, Unless differently indicated, we use a tracklet of size 8 × 224 × 224, which consists of 8 images. The patch size is set to 16 × 16 pixels. The spatial and temporal transformers share the same architecture design, with 1 layer and 6 heads. The framework of our model is implemented based on the Pytorch toolbox. The model is trained on four GPU (24G memory, produced by ASUS and sourced in China), and the whole network is updated by the algorithm of stochastic gradient descent [53] with an initial learning rate of 10^−3^, Nesterov momentum of 0.9, and weight decay of 5 × 10^−4^. The batch size is set to 32. To effectively train the model, these datasets are augmented by random cropping, random erasing, and horizontal flipping. The spatial transformer and temporal transformer share the same network structure. During inference, a single temporal sequence in the middle of the video is sampled for re-identification. In some complex scenes, where the pedestrian may be blurry due to camera proximity or angle issues, we will first enhance the pedestrian by some heuristic methods. For example, we extract the unobstructed body regions of a person based on key points of their posture. First, pose detectors are used to detect the key points of the human body. Then, based on reliable key points, a rectangular local image area is extracted from the image, which covers the human body. Therefore, each frame of the video is converted into an image that only contains the human body. Finally, this image is used as a guard to perform the attention on the original frame to enhance the pedestrian.

### 4.3. Comparison Result

In the first experiment, we compare our model with the state-of-the-art models on the three datasets MARS, iLIDS-VID, and PRID2011. The experimental result is presented in Table 2. From the table, several conclusions can be obtained. First, on most of the metrics on the three datasets, our model has the best performance. Specifically, on the dataset iLIDS-VID our model has the best performance, and our model obtains comparable or even better performance than other models on MARS and PRID2011. The result demonstrates that the fusion of different modalities of features with fine-grained cross-modal learning is effective for pedestrian re-identification.

The baseline methods mainly learn features from the temporal dimension or spatial dimension, and some others try to fuse multiple features of the global representation level. As for the first category, multi-granularity spatial cues are extracted by MGRA [7] under the guidance of a global view, and various attribute-aware features in the spatial dimension are mined by Attribute [28] for alignment. Those methods mainly focus on learning diverse spatial features and obtain remarkable performances, such as MGRA obtaining a mAP value of 85.9% on MARS. On the other hand, GLTR [32] aims to mine multi-granular temporal dependencies for short- and long-term temporal cues, and GRL [54] adapts a bi-direction temporal module to refine disentangled spatial features. Accordingly, GRL attains an excellent performance of rank 1 on the dataset iLIDS-VID. Those methods mainly focus on exploiting discriminative cues from the temporal dimension to improve recognition performance. Compared with these models, we learn appearance features from both spatial and temporal dimensions, and moreover the gait feature is also learned to complement the visual appearance feature. These two types of features are also refined and aligned to obtain a more effective representation.

**Table 2:** Comparison with the state-of-the-art models on MARS, iLIDS-VID, and PRID2011.

Models	MARS				iLIDS-VID			PRID2011		
	mAP	Rank 1	Rank 5	Rank 20	Rank 1	Rank 5	Rank 20	Rank 1	Rank 5	Rank 20
Snippet [34]	76.1	86.3	94.7	98.2	85.4	96.7	99.5	93.0	99.3	100
STAN [4]	65.8	82.3	-	-	80.2	-	-	93.2	-	-
STMP [9]	72.7	84.4	93.2	96.3	84.3	96.8	99.5	92.7	98.8	99.8
M3D [13]	74.0	84.3	93.8	97.7	74.0	94.3	-	94.4	100	-
Attribute [7]	78.2	87.0	95.4	98.7	86.3	87.4	99.7	93.9	99.5	100
PGANet [44]	81.3	87.2	94.5	96.1	82.2	94.2	99.3			
VRSTC [35]	82.3	88.5	96.5	97.4	83.4	95.5	99.5	-	-	-
GLTR [36]	78.5	87.0	95.8	98.2	86.0	98.0	-	95.5	100	-
COSAM [37]	79.9	84.9	95.5	97.9	79.6	95.3	-	-	-	-
MGRA [6]	85.9	88.8	97.0	98.5	88.6	98.0	99.7	95.9	99.7	100
STGCN [38]	83.7	89.9	-	-	-	-	-	-	-	-
AFA [39]	82.9	90.2	96.6	-	88.5	96.8	99.7	-	-	-
TCLNet [11]	85.1	89.8	-	-	86.6	-	-	-	-	-
GRL [40]	84.8	91.0	96.7	98.4	90.4	98.3	99.8	96.2	99.7	100
MTV [55]	85.8	91.1	96.9	98.7	90.9	98.2	99.8	96.0	99.5	100
TMT [31]	85.8	91.2	97.3	98.8	91.3	98.6	100	96.4	99.3	100
DCCT [32]	86.3	91.5	97.4	98.6	91.7	98.6	-	96.8	99.7	-
MTLA (Ours)	86.7	91.9	98.0	98.7	91.9	98.6	100	97.0	99.6	100

As for the second category, a spatial–temporal attention module with free-parameter method is proposed by STAN [5] to weight local features in the spatial–temporal domain. Some models design two-stream networks to learn different feature representations. M3D [10] uses 3D-CNNs and 2D-CNNs to encode spatial and temporal features, respectively. STGCN [55] adapts two parallel GCNs to mine relations from the spatial and temporal dimensions. Though DCCT [39] implements spatial complementary learning with cross-branch mutual guidance, it directly combines the global features learned by different models. Some other works try to fuse multi-view features for pedestrian re-identification. TMT [11] proposes to reassemble the spatial, temporal, and spatial–temporal features extracted by ResNet-50 [56] from the vision input. However, this method mainly fuses the global representations of different views, which is not effective for learning fine-grained semantic information and inter-view correlations. MTV [57] creates multiple input representations, or “views”, of the input, by tokenizing the video using tubelets of different sizes. Then the multiple representations are fused by a multi-view transformer. Different from these works, we learn the appearance and gait token features by divided spatial–temporal transformers and the gait recognition method, respectively. Then, these two modalities of features are aligned at the token level, which is more effective for learning semantic information from modality. Therefore, our model performs better than the state-of-the-arts approaches on most of the metrics.

**Figure 3:**
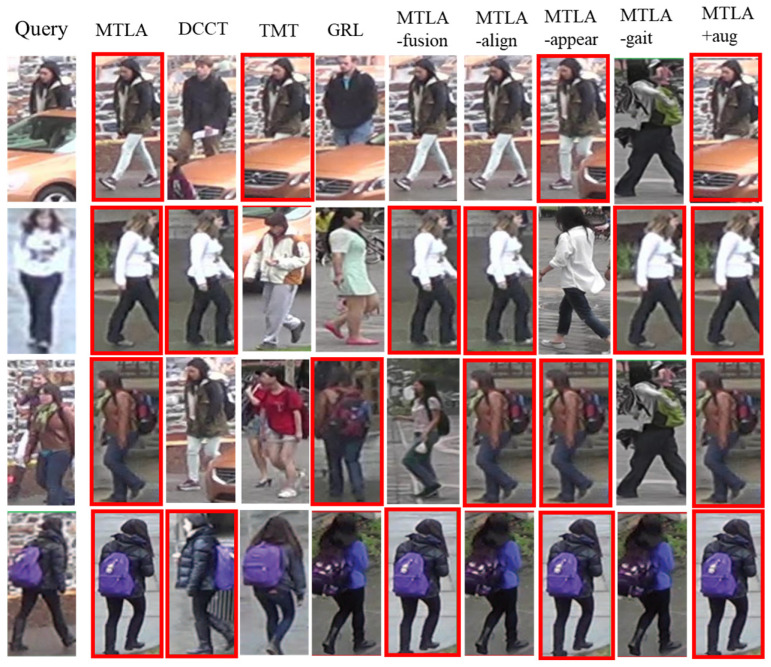
Examples of top one results of different approaches, and the images of the correct ID are marked with red boxes.

To further show the performance of different approaches, some examples of top one results retrieved based on the representations learned by different approaches are shown in Figure 3. In this figure, the query and result examples are represented by an image selected from the video sequence, and the images of the correct ID are marked with red boxes. From this figure, it is also demonstrated that our approach to learning and fusing fine-grained features from the appearance and gait modalities can improve the performance of video-based pedestrian re-identification.

### 4.4. Ablation Experiments

To further investigate the effectiveness of the MTLA, a set of ablation experiments are conducted on the three datasets. We evaluate the effectiveness of the key components by removing them from the MTLA. For example, to test the effectiveness of the discriminative feature learning of the classification loss in the feature learning, we remove it from MTLA and name it as MTLA-loss to report the performance. To test the effectiveness of token-based cross-modal alignment, we remove the alignment module to directly fuse the tokens learned from different modalities and name it as MTLA-align. To test the effectiveness of correlation-aware fusion, we combine the appearance and gait token features directly for pedestrian identification and name it as MTLA-fusion. Then, the effectiveness of token learning is also tested by feeding the model with the raw feature directly, and the models are named MTLA-appear and MTLA-gait, respectively. The ablation models are defined in Table 3.

The ablation experiment result is shown in Table 4, and some examples of the top one results retrieved by different ablation models are shown in Figure 3. From the result, it can be found that when removing different components from the MTLA, the performance is always decreased. When all the components are combined in the model, the performance is the best. Therefore, it can be concluded that all of these components contribute to the performance of pedestrian re-identification. It demonstrates that learning fine-grained denoised tokens from different modalities is effective in improving the representation. Token-based alignment and correlation-aware fusion are effective for learning the latent relation between different modalities for video-based pedestrian Re-ID. On the other hand, when we include the data augmentation process, the performance of the MTLA is improved to some degree.

### 4.5. Parameter Sensitivity

We also conduct experiments to investigate the effect of different parameter values. In our model, there are two important parameters, i.e., the length of tracklet and the depth of the transformer. Table 5 and Table 6 show the performance of the MTLA with different values for the two parameters. It can be seen that the performance is sensitive to the parameter values to some degree. Specifically, the model obtains the best performance when the tracklet length is 8 and the depth of the transformer is 2. Increasing the depth of the transformer and the length of tracklet does not improve the performance, while the model complexity is increased.

## 5. Discussion

This study is designed to deeply fuse multimodal features at the token level for video-based pedestrian re-identification. In reality, there are usually a large amount of different types of features related to a pedestrian, such as appearance, gait, attire, expression, and so on. These features reflect the characteristics of a pedestrian from different modalities, and different modalities of features supplement each other. Therefore, only one type of feature might be ineffective for re-identifying pedestrians. Accordingly, there are already some studies [10,11,39] fusing different features for pedestrian re-identification, which have achieved a certain success. However, these methods mainly regard the different types of features as features from different levels of the same model, or they fuse different types of features at the global level, which might introduce a great amount of redundant and noisy information. On the other hand, these methods fuse the features by exploiting the relation of two instances. The bipartite relation is not enough to reflect the correlation of features from different modalities.

Our method extracts multiple features from different modalities and learns denoised tokens to reflect fine-grained information. Like the word tokens in text data, the token feature is more abstract than the raw feature and finer-grained than the global feature. Compared to the traditional global feature, the learned token feature is more effective for capturing fine-grained semantic information and reducing the semantic gap between different modalities. Then, the features of different modalities are aligned based on the token level with the cross-modal contrastive learning method. Therefore, each modality of features is enhanced by learning from each other, and they are more effective in reflecting the different views of the pedestrian. Since different views of features are represented in different semantic spaces, we iteratively learn the latent correlation between them with cross-attention and aggregate them based on the correlation. Therefore, our method gives more consideration to fine-grained information and the correlation of different characteristic of the pedestrian, and thus it is more effective in fusing different modalities of features for pedestrian re-identification. In the space–time transformer module of our framework, the comparison complexity of each patch in each block is O(m + n + 2), where m is the number of patches of each frame and n is the number of frames in a video. In the future work, we will reduce the complexity of computing the correlation between different patches of the frame.

Though we mainly fuse the appearance and gait features for pedestrian re-identification in this paper, other types of features, such as expression and action, can also be fused by extending our method directly. Moreover, the framework of our method can also be used or revised in other domains that need to handle multiple-features data, such as multimodal sentiment analysis, video retrieval, video event detection, and so on. The limitation of our method is that it needs more instances of the same pedestrian, since it learns the relation of multiple instances. However, this problem can be alleviated by data enhancement methods.

## 6. Conclusions

In this paper, we propose to learn multimodal tokens and align them to obtain a fused representation for video-based pedestrian re-identification. Different from existing approaches which fuse multimodal features directly, we learn tokens from the raw features, and multimodal features are aligned at the token level. Therefore, our approach is more effective in capturing fine-grained semantic information and fusing it to obtain a comprehensive representation. Extensive experiments on three benchmarks demonstrate the superiority of our model and the effectiveness of the key components.

In future works, it would be interesting to exploit multimodal pre-training models to more effectively learn the semantics of body images. We can also extend the dual network to a triple network to fuse more modalities of feature based on fine-grained correlation for pedestrian re-identification. Moreover, this model can also be combined with other visual and gait feature extraction models for pedestrian re-identification. The work provides a rigorous academic reference for video-based pedestrian re-identification, and it can be applied to video surveillance in public places.

## Figures and Tables

**Table 1:** Statistics of datasets.

Dataset	# Tracklets	# Identities
iLIDS-VID	600	300
PRID-2011	400	200
MARS	20,478	1261

**Table 3:** Definition of ablation models.

Ablation Models	Definition
MTLA-loss	MTLA without the classification loss of the spatial and temporal transformer
MTLA-fusion	The multimodal tokens are concatenated directly without the correlation-aware fusion process
MTLA-align	The multimodal tokens are directly fed to the correlation-aware fusion procedure without alignment
MTLA-token	MTLA without the token-learning procedure, and the multimodal features learned by transform and GaitSet are directly fused for pedestrian Re-ID
MTLA + aug	Perform data augmentation before training

**Table 4:** Ablation experimental results on MARS, iLIDS-VID, and PRID2011.

Models	MARS				iLIDS-VID			PRID2011		
	mAP	Rank 1	Rank 5	Rank 20	Rank 1	Rank 5	Rank 20	Rank 1	Rank 5	Rank 20
MTLA-loss	85.6	89.2	96.4	97.9	91.0	98.2	99.7	96.0	99.3	100
MTLA-fusion	86.1	90.9	97.1	98.1	90.6	98.1	99.8	96.3	99.4	100
MTLA-align	86.0	90.1	96.6	98.0	90.4	97.6	98.6	95.9	98.8	99.6
MTLA-token	84.5	88.6	95.2	97.8	89.3	95.9	99.1	93.2	97.1	99.2
MTLA + aug	86.8	91.9	98.2	98.7	92.0	98.6	100	97.0	99.7	100
MTLA	86.7	91.9	98.0	98.7	91.9	98.6	100	97.0	99.6	100

**Table 5:** Ablation experiment for length of tracklet.

Models	MARS				iLIDS-VID			PRID2011		
	mAP	Rank 1	Rank 5	Rank 20	Rank 1	Rank 5	Rank 20	Rank 1	Rank 5	Rank 20
Length = 6	85.9	91.2	96.9	98.1	90.8	97.6	99.8	95.5	99.1	99.9
Length = 8	86.7	91.9	98.0	98.7	91.9	98.6	100	97.0	99.6	100
Length = 10	86.2	91.3	97.7	98.4	91.4	98.3	99.8	96.3	99.6	100
Length = 12	85.3	90.5	96.5	97.6	90.5	97.5	99.9	96.4	99.3	99.7

**Table 6:** Ablation experiment for depth of transformer.

Models	MARS				iLIDS-VID			PRID2011		
	mAP	Rank 1	Rank 5	Rank 20	Rank 1	Rank 5	Rank 20	Rank 1	Rank 5	Rank 20
Depth = 2	86.7	91.9	98.0	98.7	91.9	98.6	100	97.0	99.6	100
Depth = 4	86.3	91.5	97.5	98.3	91.5	98.5	100	96.4	99.1	99.9
Depth = 6	86.1	91.0	97.1	98.0	91.1	98.0	99.8	95.9	98.7	99.6

## Data Availability

All the data in this study can be generated through the presented methods.
